# The association between plant-based diet indices and metabolic syndrome: a systematic review and dose–response meta-analysis

**DOI:** 10.3389/fnut.2023.1305755

**Published:** 2024-01-08

**Authors:** Ali Nikparast, Elahe Etesami, Jamal Rahmani, Nazgoli Rafiei, Matin Ghanavati

**Affiliations:** ^1^Pediatric Gastroenterology and Hepatology Research Center, Pediatrics Centre of Excellence, Children’s Medical Center, Tehran University of Medical Sciences, Tehran, Iran; ^2^Department of Clinical Nutrition & Dietetics, Faculty of Nutrition Science and Food Technology, Shahid Beheshti University of Medical Sciences, Tehran, Iran; ^3^Department of Nutrition, Science and Research Branch, Islamic Azad University, Tehran, Iran; ^4^Cancer Research Center, Shahid Beheshti University of Medical Sciences, Tehran, Iran; ^5^Faculty of Nutrition Sciences and Food Technology, Shahid Beheshti University of Medical Sciences, Tehran, Iran

**Keywords:** plant-based diet index, metabolic syndrome, metabolic syndrome components, metaanalysis, dose–response, PDI

## Abstract

**Aim/introduction:**

The prevalence of metabolic syndrome (MetS) and its components have markedly increased worldwide. Among lifestyle factors introduced to lower the risk of MetS, healthy dietary patterns have gained considerable attention. This study aimed to assess the association between adherence to plant-based diet indices including O-PDI (overall plant-based diet index), H-PDI (healthy plant-based diet index), U-PDI (unhealthy plant-based diet index), and risk of MetS development.

**Methods:**

To find related observational studies which assessed the association between Plant-based Diet indices and risk of MetS development, PubMed/Medline, Scopus, and Web of Science databases were searched from January 2016 to November 2023. A random effects model was used to estimate pooled odds ratios (OR) and 95% confidence intervals (95% CI). To assess the heterogeneity of included studies, the *I*^2^ index was used.

**Results:**

Nine studies including 34,953 participants from the initial 288 studies were recognized to include in this meta-analysis study. According to pooled analysis, there was a significant relationship between the adherence to H-PDI and the lower risk of MetS (ES: 0.81; 95% CI: 0.67, 0.97; *I*^2^ = 77.2%, *p* < 0.001), while greater adherence to U-PDI was associated with 27% increases in the risk of MetS (ES: 1.27; 95% CI: 1.05, 1.54; *I*^2^ = 76.8%, *p* < 0.001). According to our analysis of the association between adherence to PDIs and the risk of MetS components, greater adherence to O-PDI and H-PDI was significantly associated with a higher risk of elevated FBS and obesity, respectively. As well, greater adherence to U-PDI was significantly associated with a higher risk of obesity, hypertriglyceridemia, low HDL-C, and elevated FBS.

**Conclusion:**

Our results highlighted the importance of food choices in the context of a plant-based dietary pattern, indicating that adherence to unhealthy plant-based dietary patterns rich in less healthful carbohydrates may induce the risk of MetS development.

**Systematic review registration:**

PROSPERO CRD42023428981.

## Introduction

Metabolic syndrome (MetS), known as a growing public health condition, is characterized by the presence of a cluster of metabolic abnormalities including obesity, impaired fasting blood sugar (FBS), hypertriglyceridemia, low levels of high-density lipoprotein cholesterol (HDL-C), and elevated blood pressure (BP) (1). In recent years, MetS prevalence has alarmingly increased and it has been estimated that this public health problem affects 20%–25% of the global adult population (2) not only in developed countries but also in developing ones (3–5). There is considerable evidence to suggest that MetS contributes significantly to the increased risk of cardiovascular diseases (CVDs), and all-cause mortality by 2 and 1.5 folds, respectively (6). Although the exact pathophysiology of MetS is not yet completely understood, abdominal obesity and insulin resistance, which may be caused by a sedentary lifestyle and improper dietary patterns, are likely to play a key consideration role in MetS development (2).

In this regard, it should be noted that diet is one of the important modifiable risk factors for MetS; therefore, many articles have been published regarding the provision of healthy dietary patterns for MetS prevention and management (7, 8). Several epidemiological studies have sought to determine whether diets consisting primarily of plant-based foods and a limited amount of animal products are associated with MetS, however, the results have been inconsistent (9–15). The findings of several studies have demonstrated that individuals who restricted the intake of animal-based foods (poultry, meat, fish) displayed favorable metabolic characteristics (lower body mass index (BMI), lower FBS, and lower BP) (12, 15), while others found no association (11, 13) or an adverse relationship (9, 10, 14). Accordingly, these conflicting findings may result from the fact that these studies have focused primarily on limiting animal-based foods without considering the type and quality of plant-based foods (healthy and less healthy plant foods), which may influence metabolic risk factors (12, 16–18). Concerning this, existing studies on plant-based diets and the risk of chronic disease have demonstrated that plant-based foods that are less nutrient-dense (such as refined grains, potatoes, and sugar-sweetened beverages) were significantly associated with a higher risk of obesity, type 2 diabetes, and hypertension risk (18–20).

In light of these important gaps in the literature, in 2016, innovative plant-based indices (PDIs), based on a graded scoring system for each food item, have been promoted as a strategy to align specific plant foods with health outcomes (20). PDIs consist of the following three indices: (1) overall plant-based diet index (O-PDI), representing the consumption of all plant-based foods along with decreasing consumption of animal-based foods; (2) healthful plant-based diet index (H-PDI), indicating positive scores for healthy plant-based foods (including whole grains, fruits, vegetables, nuts, legumes, vegetable oils, etc.), and reverse scores for less healthy plant-based foods (such as refined grains, potatoes, fruit juices, etc.) and animal-based foods; (3) unhealthful plant-based diet index (U-PDI), which is characterized by consumption of less healthy plant-based foods and lower consumption of healthy plant-based foods and animal-based foods.

Recently, most studies have attempted to assess the association between PDIs and odds of MetS; however, their results have been inconclusive (3–5, 21–26). It has been found that H-PDI and U-PDI were significantly associated with decreasing (22, 25) and increasing (24, 25) risk of MetS, respectively. In addition, Bhupathiraju et al. demonstrated no significant association between PDIs adherence and risk of MetS, however, adherence to the H-PDI was significantly redeuced the risk of MetS component including obesity and elevated FBS (3). Due to this inconstancy, our objective was to conduct a systematic review and meta-analysis of observational studies to investigate whether adherence to the PDIs can be associated with the risk of Mets and its related component, irrespective of currently recognized risk factors.

## Method

This systematic review and meta-analysis has been registered on the PROSPERO website (registration number: CRD42023428981) as well as it has been carried out in line with the guidelines of the Meta-analysis of Observational Studies in Epidemiology (MOOSE) (27).

### Search strategy method

To conduct a comprehensive literature search, one author (AN) conducted a thorough search of electronic databases including Scopus, PubMed/MEDLINE, as well as Web of Science from 1 January 2016 to 30 September 2023, regardless of limited to English language publications, using the combination of relevant MESH and non-MESH keywords. The time restriction is imposed due to the fact that PDIs score was developed by Satjia et al. and published in 2016 (20). Supplementary Table S1 presents the details of the electronic search strategies employed by these international databases. In addition, the references of all included publications were manually searched to ensure that no potentially relevant publications that may be missed through electronic database searches, were overlooked.

### Inclusion criteria

In our meta-analysis we included studies if they met the following criteria: (1) English language publications conducted on the adult population (aged 18 years old and above) (2) observational studies including case–control, cohort (retrospective or prospective), and cross-sectional design (3) studies including Mets cases and appropriate control groups (4) studies that have reported the association between PDIs and odds of MetS as a primary outcome of interest, and its component, including the obesity, hypertriglyceridemia, low levels of HDL-C, elevated FBS, and elevated BP, as a secondary outcome of interest.

### Exclusion criteria

Studies were excluded from the review if they were (1) review articles, (2) case reports, case series, editorials, commentaries, notes, letters with insufficient data, and conference abstracts (3) interventional studies, (4) animal research, (5) *in vivo* and *in vitro* experiments, (6) studies conducted on the infants, children, adolescents, pregnant and lactating women. Whenever more than one report was available for each of the eligible studies, the report presenting results for the most extensive time was employed.

### Data extraction and quality assessment

The relevant data for the present systematic review and meta-analysis were independently extracted by two reviewers (AN, EE), and any disagreements were clarified by consulting a senior author (JR). The first author’s name, publication year, study country, study type, study duration, number and gender of participants, mean age and BMI of participants, dietary intake method, metabolic syndrome definition criteria, variables that are taken into account for adjustments in multivariate analyses, the corresponding effect size (ES), and 95% of Confidence Interval (CI) of MetS and its component odds comparing the best (highest category) and poorest (lowest category) adherence to PDIs including O-PDI, H-PDI, and U-PDI that adjusted to account for the most confounding factors, were extracted from included studies.

For the current systematic review and meta-analysis, the quality assessment of included studies was evaluated for bias employing the Newcastle-Ottawa Scale (NOS), which is designed specifically to assess the quality of non-randomized studies (28). In accordance with this scale, the quality of the studies, in terms of the star system, is assigned based on three criteria as follows: (1) selection of the study groups (4 items), (2) comparability of the groups (2 items), and the assessment of either the exposure or outcome of interest for case–control or cohort studies, respectively, (3 items). Each item is eligible for a maximum of one star, except for the comparability item, which is eligible for a maximum of two stars. Publications with a score ≥ 7 were categorized as high quality/low risk of bias, whereas those with a score < 7 were classified as low quality/high risk of bias publications.

### Statistical analysis

Statistical analyses were carried out using STATA 14.0 (Stata Corporation, College Station, Texas, United States), as well as a *p*-value < 0.05 was regarded as a significant level. As a starting point for calculating the ES of MetS and its components, we considered the lowest category of PDIs as a reference category (29). The Mets and its components’ effect size estimates were pooled using the DerSimonian and Laird random-effects model (30). In order to evaluate heterogeneity between studies, the Cochran Q test (P heterogeneity) and the *I*^2^ statistic were employed. Whenever there was heterogeneity, the significance level was set at *p* ≤ 0.10 for Cochran Q. According to *I*^2^ metrics, heterogeneity of 25%, 50%, and 75% is indicative of low, medium, and high heterogeneity, respectively. To find the potential source of the heterogeneity, subgroup analysis using the inverse-variance fixed effects model for pooling ES of MetS (30), as well as meta-regression analysis were conducted. In addition, the curvilinear dose–response association between PDIs and the effect size of metabolic syndrome was also assessed using restricted cubic splines with three knots at fixed percentiles of 10th, 50th, and 90th (31). Nonlinearity was evaluated by evaluating the *p* value of the coefficient at the second spline. An evaluation of publication bias among studies was conducted using visual inspection of funnel plots and Egger’s and Begg’s regression asymmetry test (32).

## Results

### Study selection

**Figure 1:**
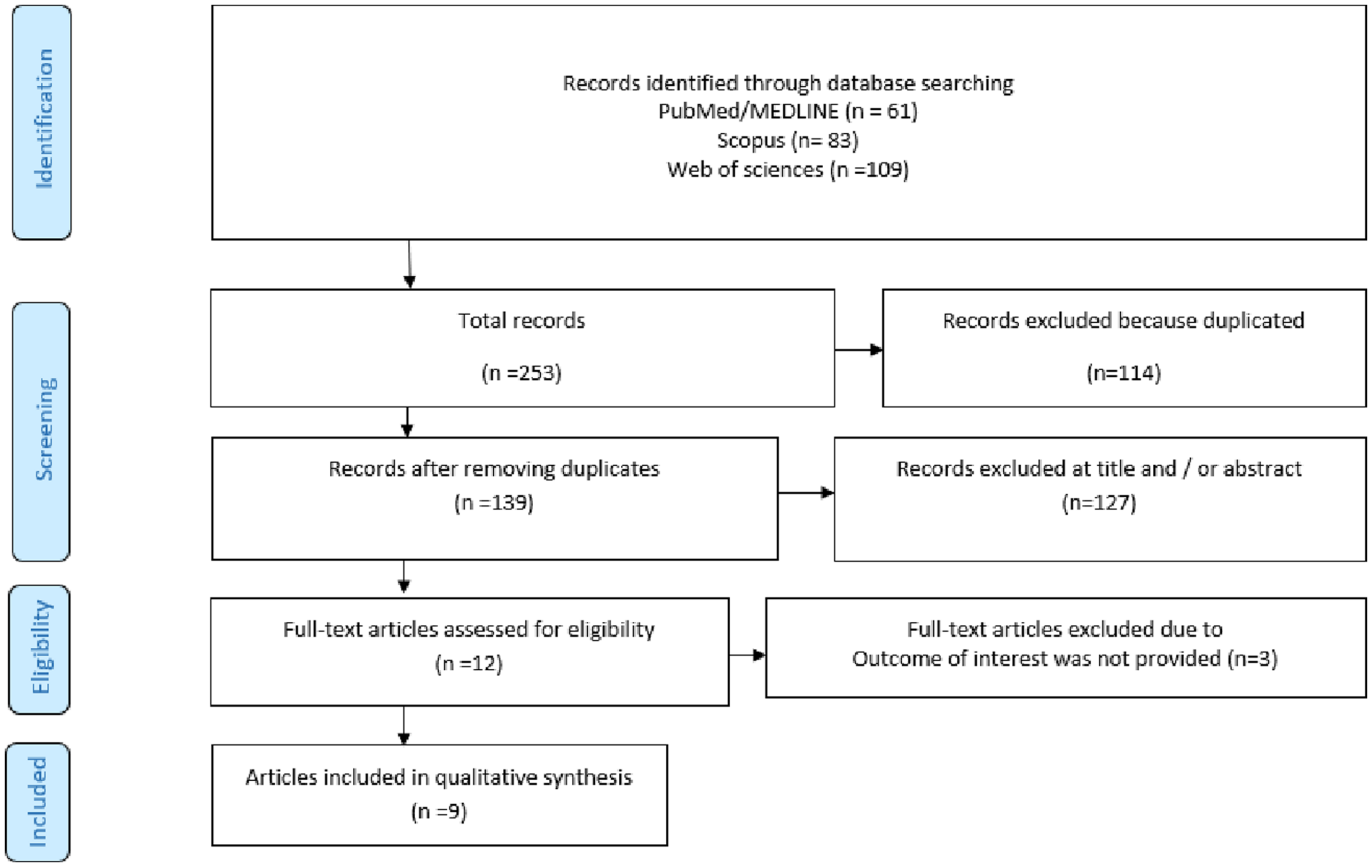
Flow chart of included studies.

An overview of the selection process is provided in Figure 1 accompanied by references retrieved from the electronic databases. A total of 253 relevant papers were identified during the preliminary literature search of electronic databases, including PubMed/MEDLINE (*n* = 61), Scopus (*n* = 83), and ISI Web of Science (*n* = 109). Following the removal of duplicate papers (*n* = 114) and studies that were not relevant as determined by title and abstract screening, 12 potentially relevant papers have been selected to undergo full-text review. Upon assessing the eligibility of the remaining papers according to our research topic, 3 papers were excluded due to the lack of outcome of our interest; finally, 9 relevant papers were included in the current systematic review and dose–response meta-analysis (3–5, 21–26).

### Study characteristics

Table 1 describes the characteristics of eligible studies [including 7 cross-sectional studies (3, 5, 21, 22, 24–26) and 2 cohort studies (4, 23)], which included a total of 34,953 participants. The eligible studies have been conducted in Iran (5, 21, 22, 26), South Korea (23, 24), China (4), Denmark (25), and the United States (3). These studies were published between 2020 and 2023. The mean age and BMI of the participants ranged from 40.8 to 67 years and 19 to 32.6, respectively. Dietary intakes were assessed in seven studies using the food frequency questionnaire (3, 5, 21–24, 26), as well as in two studies using a 24-h recall (4, 25). Among the 9 included studies, MetS was defined according to the National Cholesterol Education Program Adult Treatment Panel III (NCEP ATP III) in 3 studies (21–23), the Joint Interim Statement (JIS) in 4 studies (5, 24–26), and the National Chinese guidelines in 1 study (4). However, 1 study did not provide the method by which MetS was defined (3).

**Table 1:** Main characteristics of included studies.

References	Country	Study type (median of follow-up)	Population (male/female)	Case	Mean age	Mean BMI	Dietary assessment method	Diagnosis criteria for the metabolic syndrome	Adjustment for confounders	Quality score
Kim et al. (23)	South Korea	Cohort (8 years)	5,646 (2,952/26,94)	2,583	50.9	23.8	FFQ	NCEP ATP III	Age, sex, BMI, PA, smoking status, educational level, alcohol intake, and total energy intake,	8
Amini et al. (21)	Iran	Cross-sectional	178 (51/127)	95	67	29.7	FFQ	NCEP ATP III	Age, sex, BMI, PA, smoking status, marital status, Type 2 diabetes, hypertension, dyslipidemia, and total energy intake	10
Kim et al. (24)	South Korea	Cross-sectional	14,450 (5,585/8,865)	3,371	41.1	23.5	FFQ	JIS	Age, sex, BMI, PA, smoking status, educational level, income level, alcohol intake, and total energy intake.	10
Bhupathiraju et al. (3)	United States	Cross-sectional	891 (472/419)	306	61.4	26	FFQ	-	Age, sex, BMI, PA, smoking status, educational level, alcohol intake, study site, family history of diabetes, years lived in the United States, diabetes medication use, cholesterol-lowering medication, hypertension medication use, sum of cultural traditional measures, and total energy intake	7
Huo et al. (4)	China	Cohort (5 years)	10,013 (5,272/4,741)	961	46.9	19	24-h recall	National Chinese guidelines	Age, sex, PA, smoking status, educational level, alcohol intake, total energy intake, total carbohydrate intake, total fat intake, and total protein intake	8
Jafari et al. (22)	Iran	Cross-sectional	2,225 (1,187/1,038)	607	45.5	26.6	FFQ	NCEP ATP III	Age, BMI, PA, smoking status, educational level, marital status, menopausal status, and medication use	10
Shahdadian et al. (26)	Iran	Cross-sectional	527 (286/241)	151	42.6	26	FFQ	JIS	Age, sex, BMI, PA, smoking status, educational level, socio-economic status, marital status, Total energy intake, margarine, and hydrogenated oil	
Vajdi et al. (5)	Iran	Cross-sectional	347 (202/145)	142	40.8	32.6	FFQ	JIS	Age, Sex, PA, smoking status, educational level, occupation, marital status, and total energy intake	9
Lanuza et al. (25)	Denmark	Cross-sectional	676 (305/371)	155	48	24.5	24-h recall	JIS	Age, sex, PA, time point, smoking status, alcohol intake, and total energy intake	9

BMI, Body Mass Index; FFQ, Food Frequency Questionnaire; JIS, Joint Interim Statement; NCEP ATP III, National Cholesterol Education Program Adult Treatment Pane III; PA, Physical Activity.

Table 2 shows the quality assessment of the included studies using the NOS. In the current meta-analysis, all the included studies were high in quality.

**Table 2:** Quality assessment of studies included in this study on the PDIs and risk of MetS.^a^

Study	Selection	Comparability	Outcome	NOS score
Kim H et al.	****	**	**	8
Amini MR et al.	*****	**	***	10
Kim H et al.	*****	**	***	10
Bhupathiraju SN et al.	****	**	*	7
Huo Y et al.	****	**	**	8
Jafari F et al.	*****	**	***	10
Shahdadian F et al.	***	**	***	8
Vajdi M et al.	****	**	***	9
Lanuza F et al.	****	**	***	9

*One point.

a According to the Newcastle-Ottawa Scale (NOS) criteria (8).

### Finding from the meta-analysis

#### O-PDI and MetS, and it’s related components

Table 3 represents the overall multi-variable adjusted effect sizes (ES) from the random-effect meta-analysis of O-PDI and odds of MetS and its related components. Pooling 8 study estimates indicated no significant association between the greater adherence to O-PDI and odds of MetS (ES: 0.97; 95% CI: 0.89, 1.06), with low heterogeneity between studies (*I*^2^ = 12.6%, P-heterogeneity = 0.33). Table 4 indicates subgroup analysis of the association between OPDI and odds of MetS. Subgroup analysis showed that region, dietary assessment tools, BMI adjustment were all potential sources of heterogeneity. Subgroup analysis based on region and dietary assessment tools revealed that one study used food recall as tools and conducted in Europe showed a significant association between greater adherence to OPDI and odds of MetS. Moreover, studies controlled for BMI as confounder in their multi-variates adjusted models failed to show any significant association between OPDI and MetS.

**Table 3:** Plant-based diet indices in relation to metabolic syndrome and its related components based on analysis of the highest compared with lowest adherence.

	Study estimates, *n*	Effect size (95% CI)	*I*^2^ (%)	P-heterogeneity
**Overall plant-based diet index**				
Metabolic syndrome	8	0.97 (0.89–1.06)	12.6	0.33
Obesity	6	0.90 (0.78–1.04)	38	0.15
Hypertriglyceridemia	5	0.97 (0.87–1.09)	0	0.46
Low HDL-C	5	0.93 (0.71–1.21)	60.7	0.04
Elevated fasting blood sugar	5	**0.85 (0.76–0.94)**	0	0.55
Elevated blood pressure	6	0.97 (0.89–1.05)	0	0.93
**Healthy plant-based diet index**				
Metabolic syndrome	9	**0.81 (0.67–0.97)**	77.2	<0.001
Obesity	7	**0.83 (0.69–0.99)**	73.4	<0.01
Hypertriglyceridemia	6	0.94 (0.84–1.04)	0	0.45
Low HDL-C	6	0.87 (0.68–1.10)	39.7	0.14
Elevated fasting blood sugar	6	0.89 (0.77–1.03)	29.7	0.21
Elevated blood pressure	7	0.94 (0.88–1.01)	0	0.95
**Unhealthy plant-based diet index**				
Metabolic syndrome	9	**1.27 (1.05–1.54)**	76.8	<0.001
Obesity	8	**1.31 (1.09–1.59)**	81.2	<0.001
Hypertriglyceridemia	7	**1.26 (1.13–1.41)**	18.6	0.29
Low HDL-C	7	**1.24 (1.14–1.35)**	0	0.77
Elevated fasting blood sugar	7	**1.12 (1.02–1.23)**	2.4	0.41
Elevated blood pressure	8	1.08 (0.95–1.22)	58	0.02

CI, confidence interval; HDL-C, high-density lipoprotein cholesterol.

Bold values represent statistical significance at the *p*<0.05 level.

**Table 4:** Subgroup analysis of the association between plant based dietary indices and metabolic syndrome.

Characteristics		Studies, No	Effect size (95% CI)	*I*^2^ (%)	*p* value for heterogeneity between subgroups
**Overall plant-based diet index**					
**Study type**					
Cohort studies		1	0.96 (0.82–1.04)	-	0.81
Cross-sectional studies		7	0.98 (0.89–1.07)	24.6	
**Region**					
United states		1	0.97 (0.85–1.10)	-	**0.05**
Europe		1	**0.47 (0.26–0.85)**	-	
Asia		7	0.99 (0.90–1.08)	0	
**Age**					
<45		3	0.96 (0.81–1.15)	0	0.93
≥45		5	0.97 (0.90–1.05)	49.4	
**Sex**					
Male		3	0.91 (0.73–1.13)	54	0.25
Female		3	1.08 (0.88–1.33)	50.6	
**Body mass index**					
Normal		3	0.94 (0.85–1.04)	62.8	0.35
Overweight or obese		5	1.01 (0.90–1.13)	0	
**Number of participants**					
≤5,000		6	0.98 (0.88–1.10)	36.7	0.74
>5,000		2	0.96 (0.87–1.06)	0	
**Dietary assessment tools**					
Food frequency questionnaire		7	0.98 (0.91–1.06)	0	**0.01**
Food recall		1	**0.47 (0.26–0.85)**	-	
**Adjustment for confounders**					
Body mass index	Yes	6	0.98 (0.91–1.06)	0	**0.05**
	No	2	**0.59 (0.36–0.99)**	54.8	
Alcohol consumption	Yes	4	0.95 (0.88–1.03)	45.5	0.13
	No	4	1.14 (0.91–1.43)	0	
**Healthy plant-based diet index**					
**Study type**					
Cohort studies		2	0.92 (0.82–1.02)	76.7	0.78
Cross-sectional studies		7	0.94 (0.86–1.02)	80.5	
**Region**					
United States		1	0.98 (0.88–1.09)	-	<0.001
Europe		1	**0.26 (0.14–0.49)**	-	
Asia		7	0.92 (0.84–1.00)	67	
**Age**					
<45		3	**0.90 (0.84–0.97)**	81.7	0.04
≥45		6	1.11 (0.92–1.34)	49.1	
**Sex**					
Male		4	**0.97 (0.95–0.99)**		0.93
Female		4	**0.97 (0.95–0.99)**		
**Body mass index**					
Normal		4	0.97 (0.88–1.07)	65.2	0.36
Overweight or obese		6	**0.90 (0.82–0.99)**	65.6	
**Number of participants**					
≤5,000		6	**0.89 (0.81–0.98)**	80.1	0.21
>5,000		3	0.97 (0.88–1.07)	76.4	
**Dietary assessment tools**					
Food frequency questionnaire		7	0.96 (0.90–1.03)	58.4	<0.01
Food recall		2	**0.62 (0.49–0.79)**	88.5	
**Adjustment for confounders**					
Body mass index	Yes	6	0.96 (0.90–1.03)	65.3	<0.01
	No	3	**0.64 (0.87–0.99)**	78.8	
Alcohol consumption	Yes	5	0.96 (0.89–1.03)	84.2	<0.01
	No	4	**0.68 (0.54–0.85)**	0	
**Unhealthy plant-based diet index**					
**Study type**					
Cohort studies		2	**1.41 (1.26–1.58)**	0	<0.001
Cross-sectional studies		7	**1.13 (1.03–1.25)**	76.9	
**Region**					
United States		1	0.98 (0.86–1.12)	-	<0.001
Europe		1	**2.70 (1.50–4.85)**	-	
Asia		7	**1.36 (1.24–1.48)**	49.1	
**Age**					
<45		3	**1.51 (1.26–1.81)**	0	0.01
≥45		6	**1.19 (1.10–1.29)**	82.2	
**Sex**					
Male		3	**1.28 (1.04–1.59)**	0	0.74
Female		3	**1.35 (1.12–1.63)**	82.4	
**Body mass index**					
Normal		4	**1.44 (1.30–1.58)**	0	<0.001
Overweight or obese		6	1.00 (0.90–1.12)	0.36	
**Number of participants**					
≤5,000		6	**1.44 (1.31–1.59)**	0	0.03
>5,000		3	1.02 (0.91–1.14)	58.5	
**Dietary assessment tools**					
Food frequency questionnaire		7	**1.21 (1.12–1.31)**	78	0.14
Food recall		2	**1.45 (1.16–1.81)**	80	
**Adjustment for confounders**					
Body mass index	Yes	6	**1.22 (1.12–1.32)**	81.5	0.18
	No	3	**1.42 (1.15–1.77)**	64.4	
Alcohol consumption	Yes	5	**1.27 (1.18–1.38)**	86.1	0.03
	No	4	0.98 (0.79–1.23)	0	

Bold values represent statistical significance at the *p*<0.05 level.

Also, a significant association was found between the O-PDI and odds of elevated FBS (ES: 0.85; 95% CI: 0.76, 0.94; *I*^2^ = 0.0%, p-heterogeneity = 0.55) (Table 3). However, no significant association between the O-PDI and the odds of obesity, hypertriglyceridemia, low HDL-C, and elevated BP was observed (Table 3).

#### H-PDI and MetS and its related components

Pooling 9 study estimates indicated a significant inverse association between greater adherence to H-PDI and odds of MetS (ES: 0.81; 95% CI: 0.67, 0.97), with substantial heterogeneity between studies (*I*^2^ = 77.2%, P-heterogeneity = <0.001) (Table 3). Our subgroup analysis demonstrated that the region, age, dietary assessment tools, and controlling for BMI and alcohol consumption as a confounder were all potential sources of the heterogeneity (all *p* values for heterogeneity between subgroups <0.05) (Table 4). According to our subgroup analysis stratified by region, one study which conducted in Europe demonstrated a significant inverse association between greater adherence to H-PDI and odds of MetS (ES: 0.26; 95% CI: 0.14, 0.49) (Table 4). The association between greater adherence to H-PDI and odds of MetS was only significant in individuals who were younger (<45 years old) and were overweight or obese (Table 4). As well, in studies that controlled for the BMI and alcohol consumption, no significant association between the H-PDI and odds of MetS was found (Table 4).

As well, a significant association was observed between greater adherence to H-PDI and odds of obesity (ES: 0.83; 95% CI: 0.69, 0.99; *I*^2^ = 73.4%, p-heterogeneity = <0.001) (Table 3). However, no significant association between the H-PDI and the odds of hypertriglyceridemia, low HDL-C, elevated FBS, and elevated BP was observed (Table 3).

#### U-PDI and MetS and its related components

Pooled ES of 9 studies indicated that the association between adherence to H-PDI and odds of MetS was significant (ES: 1.27; 95% CI: 1.05, 1.54), with substantial heterogeneity between studies (*I*^2^ = 76.8%, P-heterogeneity = <0.001) (Table 3). Our subgroup analysis showed that study type, region, age, BMI, number of participants, and controlling for alcohol consumption as a confounder were all potential sources of heterogeneity (all *p* values for heterogeneity between subgroups <0.05) (Table 4). Our subgroup analysis demonstrated that these association was only significant in individuals with normal BMI, and studies with sample size less than 5,000, and not controlled for alcohol consumption as a confounder (Table 4). Furthermore, these results were independent of study type, age, sex, dietary assessment tools and controlling for the BMI as a confounder (Table 4).

As well, there was a meaningful association between greater adherence to U-PDI and odds of the MetS components, except for elevated BP (Table 3).

#### Findings from the dose–response meta-analysis

**Figure 2:**
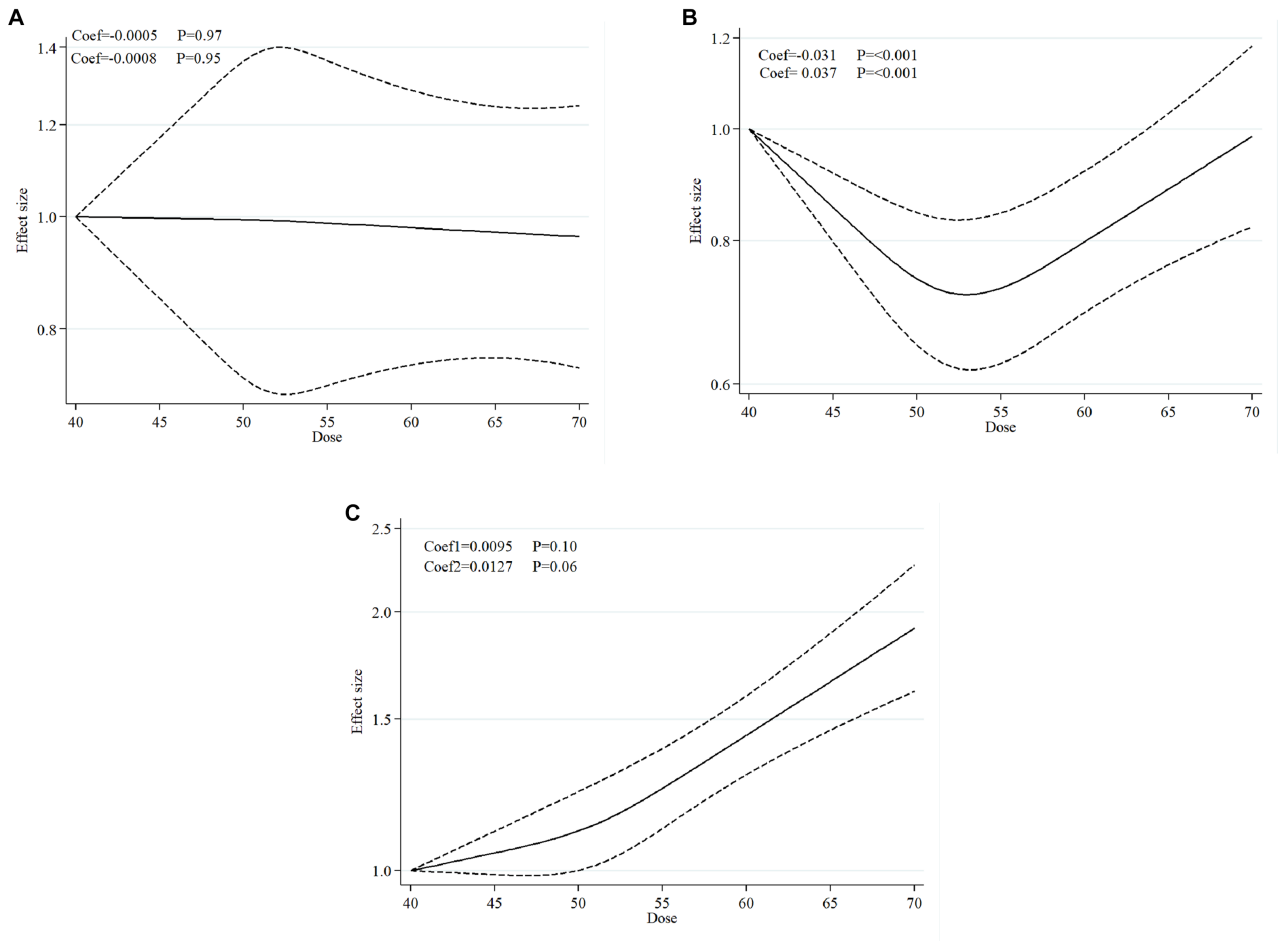
Dose-response curves from the random-effect meta-analysis of plant-based diet indices and odds of metabolic syndrome. **(A)** The dose-response association between overall plant-based diet index and odds of metabolic syndrome. **(B)** The dose-response association between healthy plant-based diet index and odds of metabolic syndrome. **(C)** The dose-response association between unhealthy plant-based diet index and odds of metabolic syndrome.

Dose–response curves from the random-effect meta-analysis of plant-based diet indices and odds of MetS is provided in Figure 2. The dose–response association between O-PDI and odds of MetS is provided in Figure 2A. The pooled effect sizes from the nonlinear dose–response model represented a non-significant association between O-OPDI and MetS (coef1 = −0.0005, *p* = 0.97; coef2 = −0.0008, *p* = 0.95). According to our findings, there was a U-shape association between H-PDI and MetS (coef1 = −0.031, *p* < 0.001; coef2 = 0.037, p < 0.001; Figure 2B) and direct association between U-PDI and odds of MetS (coef1 = 0.0095, *p* = 0.10; coef2 = 0.0127, *p* = 0.06; Figure 2C).

### Findings from meta-regression and assessment of publication bias

The meta-regression association between greater adherence to PDIs and odds of MetS based on age and BMI is provided in Supplementary Figures S1, S2, respectively. According to these findings, age and BMI was not significant sources of heterogeneity in the association between greater adherence to PDIs and odds of MetS (All *p*-values>0.05).

The assessment of Publication bias is provided in Supplementary Figure S3 demonstrating the funnel plots of ES for PDIs and odds of MetS without asymmetry and Egger’s and Begg’s tests. According to inspecting the funnel plot visually, no evidence of publication bias was observed, also confirmed by Egger’s and Begg’s tests (All p-values>0.05).

## Discussion

Developing healthy dietary patterns play an undeniable role in decreasing the probability of having MetS and its components. In this context, this is the first study evaluated the association between the novel plant-based diet scores (namely O-PDI, H-PDI, and U-PDI) and odds of MetS and its components. Therefore, we conducted this systematic review and meta-analysis for the first time, involving 9 studies and 34,953 participants, to determine whether adherence to the novel plant-based diet scores (namely O-PDI, H-PDI, and U-PDI), are associated with odds of MetS and its components. The results of our investigation demonstrated that extreme adherence to O-PDI and H-PDI was found to be associated with a lower risk of MetS. However, it is important to note that the association between O-PDI and MetS risk did not reach statistical significance. It was also found that extreme adherence to U-PDI was significantly associated with a 27% increase in likelihood of developing MetS. In subgroup analysis based on gender, age and BMI, adherence to H-PDI and reducing the odds of MetS was independent of sex differences. However, this association was only significant among younger adults and individuals who were overweight or obese. As well, the pooled analysis of studies that included BMI and alcohol consumption in their adjustment models failed to show any significant association between H-PDI and lowered risk of MetS. Furthermore, extreme adherence to U-PDI significantly increased the odds of MetS independent of age, and sex. However, this association was only meaningful among individuals with normal BMI. Furthermore, this association was found to be contingent upon alcohol intake. According to our findings regarding the relationship between adherence to PDIs and risk of MetS components, extreme adherence to O-PDI and H-PDI was associated with a lower risk of elevated FBS and obesity, respectively. As well, extreme adherence to U-PDI associated with a higher risk of obesity, hypertriglyceridemia, low HDL-C, and elevated FBS.

There is a substantial body of evidence, particularly from studies performed on Western societies, suggesting that O-PDI and H-PDI may decrease the risk of cardiometabolic diseases (8, 33, 34). A recent systematic review and meta-analysis, conducted on 307,099 participants who primarily lived in Western nations, showed that individuals who were highest adherence to plant-based dietary pattern compared to lowest ones had a 23% lower risk of type 2 diabetes. When healthy plant-based dietary pattern, including whole grains, fruits, vegetables, legumes, and nuts consumption, is used in the definition of plant-based diet, the corresponding association is even further strengthened to 30% (33). In addition, the results of a meta-analysis study conducted on longitudinal investigations including 698,707 participants who predominantly lived in the United States and the United Kingdoms indicated that individuals who were in the highest categories of plant-based diets compared to those in the lowest ones had a 14% lower risk of cardiovascular diseases (35). In recent years, several studies have been conducted to demonstrate whether adherence to PDIs is associated with MetS and its related components (3, 4, 22, 24, 26). Huo et al. performed a longitudinal study among 9,949 Chinese adults in the framework of the China Health and Nutrition Survey, which demonstrated that throughout a five-year follow-up period, participants who were in the highest quintile of H-PDI compared to those in the lowest quintile of H-PDI had a 28% lower risk of MetS after controlling for cardiometabolic and lifestyle confounders (4). However, it should be mentioned that this study did not evaluate the association between O-PDI and the risk of MetS. In addition, Jafari et al. conducted a cross-sectional study in 2,225 healthy Iranian participants in order to evaluate the association between PDIs and odds of MetS (22). According to the study’s findings, there was a nonsignificant direct association between O-PDI and odds of MetS. As well, extreme adherence to H-PDI after adjusting for a variety of potential confounders was significantly associated with a 33% decrease in odds of MetS. In addition, several studies were conducted not only in Western countries but also in Asian population demonstrated that participants who were in the highest category of H-PDI score had favorable anthropometric and cardiometabolic parameters status compared to those in the first category of H-PDI (3, 5, 21). However, Kim et al., in framework of the Korea National Health and Nutrition Examination Survey including 14,450 participants, showed that after controlling for potential confounders, no significant association was found between O-PDI and H-PDI and odds of MetS (24). Additionally, Shahdadian et al., in a cross-sectional study among 527 Iranian adults, revealed that there was a nonsignificant association between greater adherence to O-PDI and H-PDI and lower odds of MetS (26).

Even though a number of studies have been conducted on the association between adherence to O-PDI and H-PDI and odds of MetS, the findings are inconclusive. Therefore, it is important to consider several factors when interpreting the null association between O-PDI and the reduced risk of MetS. The first thing to note is that 7 out of 9 included studies were conducted in the Asian population which already tend to have lower consumption of animal-based products (36) and higher intake of plant-based foods, including whole or refined grains, potatoes, legumes, fruits, and vegetables (37). As well, one study conducted by Bhupathiraju et al. evaluated the association between PDIs and cardiometabolic risk factors among the South Asian American population living in the United States who follow a predominantly plant-based diet in accordance with their cultural and religious traditions (3). To put it another way, it would appear that studies evaluating the association between adhering to PDIs and risk of MetS were largely carried out on participants who already tend to consume plant-based dietary patterns. Therefore, variations in dietary patterns among these populations, as measured by O-PDI, may exhibit less conspicuous disparities compared to those observed in Western population which may have impaired the ability of O-PDI score in predicting odds of damaged metabolic responses. As mentioned above, Shahdadian et al. failed to demonstrate that a greater adherence to H-PDI could significantly reduce odds of MetS, however, according to their sensitivity analysis, after excluding participants who consumed fruit and vegetables greater than 1 kilogram per day, an extreme adherence to the H-PDI significantly reduced odds of MetS by 70% (26). In this context, our study provides new insights into the higher level of compliance with plant-based diets and how prioritizing the quality of plant-based foods could potentially have a beneficial effect on the likelihood of developing MetS in the Asia region that regularly adheres to plant-based dietary patterns. However, further studies are needed to evaluate the impact of adherence to O-PDI and H-PDI on odds of MetS while considering participant’s habitual dietary intakes influenced by traditional or cultural factors.

In line with our dose–response analysis, it seems that there is a U-shaped association between adherence to H-PDI and odds of MetS, implying the lowest odds of Mets were in the intermediate range of H-PDI scores. In addition, it is more likely to see a higher risk of MetS in both individuals with highest and lowest H-PDI scores. In line with our findings, Kim et al. in a framework of a community-based longitudinal study with over a median of 8 years follow-up, indicated a significant U-shape association between adherence to H-PDI and risk of MetS (23). In addition, the researchers concluded that only individuals in the fourth compared to the lowest quintile of H-PDI exhibited a significant association between adherence to H-PDI and decreased risk of MetS. Moreover, Shahdadian et al. demonstrated that there is a significant non-linear association between H-PDI and odds of MetS (26). According to these results, it seems that participants who had moderate H-PDI scores were more likely to have a lower risk of developing MetS compared to those with lowest or highest H-PDI scores. To interpret the causal mechanisms underlying the association between extreme adherence to H-PDI and increased odds of MetS, a number of plausible explanations should be mentioned. In this regard, extreme compliance with H-PDI might result in micronutrient insufficiency (23, 38). According to our included studies, it has been proposed that extreme adherence to H-PDI may not provide enough dietary calcium intake to meet the recommended dietary allowance (RDA) among adults (ranges from 1,000 to 1,200 mg per day), which can lead to an increased risk of MetS (23, 38). In this respect, Kim et al. showed that participants who were in the lowest H-PDI categories as well as those in the highest ones, received 249 and 234 mg/1000 kcal of calcium from their diets, respectively (23). In addition, in contrast to the Dietary Guidelines for Americans which suggest a minimum intake of 8 ounces of fish and seafood products per week, Bhupathiraju et al. showed that individuals who adhered more closely to the H-PDI consumed fish and seafood products less than one serving per week (3). According to the findings of a systematic review study, greater fish and seafood product consumption may have a protective effect against the likelihood of MetS (39). In addition, the presence of heavy metals and chemical pesticides in plant-based foods, particularly vegetables, which is a known global concern correlated with industrialization, may attenuate or modify the association between different PDIs and odds of MetS (5, 26, 40). In this regard, it can be assumed that maintaining a moderate level of adherence to H-PDI is associated with a decreased risk of MetS.

According to our subgroup analysis, the association between adherence to H-PDI and odds of MetS only remained significant among participants younger than 45 years old. In line with our findings, Huo et al., in the framework of a cohort study with a median follow-up of 5 years, showed a relationship between adherence to H-PDI and a reduced risk of MetS continued to be statistically significant among those who were younger than 40 years old at the baseline of their study (4). It is plausible that the observed variations in our results among age subcategories can be attributed to the different dietary habits of older and younger adults. According to the included studies, as participants got older, they tended to follow a healthier dietary pattern, which may have limited the ability to show substantial differences in dietary intakes captured by H-PDI among older than younger adults (3, 24, 26). Therefore, this particular characteristic may provide an explanation for the impaired ability of H-PDI score in reducing odds of MetS among older adults. Although it is mentioned that the prevalence of MetS appears to increase with advancing age, which is closely associated with higher risk of cardiometabolic diseases, low-grade systemic inflammation, and dysregulated metabolic pathways. Therefore, by considering the mentioned risk factors, solely adherence to H-PDI in older adults could not lead to a significant clinical response for reducing the risk of MetS. This means that following the H-PDI in older adults might not be enough to significantly lower risk of MetS (7, 41). Additional research is required to assess the correlation between adherence to H-PDI and risk of developing MetS throughout various life stages.

The findings of our study indicate that adhering to H-PDI among overweight or obese participants may potentially mitigate odds of developing MetS, however, no such association was observed among individuals who were normal BMI. There appears to be a correlation between those who are obese and their tendency to adopt a less healthy dietary pattern in comparison to those with a normal weight (5). Consequently, it is more likely that following H-PDI in obese and overweight individuals is associated with a reduced odd of MetS. These findings should be interpreted with caution due to the small number of studies. Therefore, a greater number of investigations need to be conducted to determine the role of weight status on the association between PDIs and the odds of MetS. As well, we found that the inverse association between H-PDI and odds of MetS became nonsignificant after controlling for BMI as a confounder. In this context, Hou et al. demonstrated that BMI at baseline served as a mediator for 27.8% of the relationship between adherence to H-PDI and incidence of MetS (4). It seems that BMI have potential to consider as an independent risk factor when evaluating the association between H-PDI and odds of MetS.

Our subgroup analysis revealed that the inverse association between H-PDI and odds of MetS no longer remained statistically significant when adjusting for alcohol intake as a confounding variable. In this context, the results of a meta-analysis evaluating the association between alcohol consumption and risk of metabolic syndrome showed that excessive alcohol consumption may be linked to a heightened risk of MetS, whereas minimal alcohol consumption appeared to be associated with a decreased risk of MetS (42). Furthermore, another meta-analysis demonstrated that consuming alcohol at levels below 40 g/day in males and 20 g/day in women had a notable impact on reducing the risk of MetS (43). However, it is important to note that caution should be taken when interpreting this finding due to the majority of studies included in our meta-analysis involve populations who abstain from alcohol owing to religious convictions or did not provide accurate information about their alcohol intake.

The presence of low-grade inflammation, obesity, dyslipidemia, insulin resistance, and high blood pressure are the hallmarks of the MetS (1). The possible biological and nutritional factors explaining the association between H-PDI and the lower risk of developing MetS may be as follows: lower intake of animal-based foods and higher consumption of healthy plant-based foods including whole grains, fruits, vegetables, legumes, nuts, and vegetable oils which claimed to accompany with lower levels of appetite, total energy intake, saturated fatty acid (SFA) and trans fatty acid (TFA), added sugar, sugar-sweetened foods, glycemic index, glycemic load, salty foods, as well as higher consumption of antioxidants, phytochemicals, polyphenols and isoflavones, vitamins, soluble and insoluble dietary fibers, which are all inversely associated with MetS and its components (5, 12, 22, 44).

According to our analysis of the association between O-PDI and risk of MetS components, greater adherence to O-PDI was significantly associated with 15% lower odds of elevated FBS. In line with our findings, the results of a systematic review and meta-analysis study evaluating the association between adherence to plant-based dietary patterns and the risk of Type 2 diabetes mellitus (T2DM) revealed a significant association between higher adherence to O-PDI and a 23% reduction in the likelihood of developing T2DM (33). In addition, according to this study’s dose–response analysis, there was a significant linear association between substituting plant-based foods for animal-based food products and reducing the risk of developing type 2 diabetes. Furthermore, our analysis demonstrated that extreme adherence to H-PDI significantly reduced odds of obesity by 17%. In agreement with our findings, a systematic review study aimed to evaluate the association between adherence to plant-based dietary patterns and risk of obesity showed that greater adherence to H-PDI was inversely associated with favorable weight management (45). It seems that greater adherence to H-PDI is accompanied by a higher intake of low-energy-dense and high-fiber foods, which may lead to a lower risk of obesity (4, 45).

The results of our pooled analysis revealed that extreme adherence to U-PDI was associated with a 1.27-fold increase in the odds of MetS. Our findings regarding U-PDI and MetS are largely consistent with those studies conducted in Western populations highlighting the negative health effects of U-PDI (20, 34). There are several mechanisms by which the positive association between U-PDI and MetS may be explained. Likewise, greater adherence to U-PDI would result in higher total energy intake and increased consumption of unfavorable nutrients (including simple sugars, saturated and trans fatty acids, and sodium), as well as food components (including refined grains, sugar-sweetened ones, salty ones, deep fried snacks, etc.), and a decreased consumption of micronutrients (vitamins, minerals, antioxidants, etc.), which could have unfavorable effects on the risk of MetS and its components (7, 16, 24, 44).

The results of our dose–response analysis on the association between adherence to U-PDI and odds of MetS revealed that greater adherence to U-PDI was directly associated with an increased risk of MetS. In line with our findings, Kim et al. demonstrated that after controlling the potential confounders, per 1-SD increase in the U-PDI score, was significantly associated with a 15% increase in the risk of MetS (23).

According to our gender-specific subgroup analysis, we found that extreme adherence to U-PDI was significantly associated with higher odds of Mets, independently of gender. However, this association was more strongly in men compared to women. In line with our findings Kim et al. showed that there is no sex difference in the association between extreme adherence to U-PDI and odds of MetS, as well women tend to have a stronger association between adherence to U-PDI and MetS likelihood as compared to men (24). These observed sex differences may attribute to a wide range of causes, including variations in biological elements (for example, sex hormones), lifestyle factors (dietary patterns, physical activity), genetic background, and disease management (6, 24). In addition, our age-specific subgroup analysis demonstrated that, independent of age, extreme adherence to U-PDI was significantly associated with higher odds of MetS. However, this association was more pronounce in younger adults compared to older ones. It is important to mention that, as the U-PDI score increased, the mean age of participants significantly decreased (3, 24, 26). In other words, it seems that younger adults are more likely to follow unhealthy plant-based patterns which may lead to an increase in the odds of MetS (24).

In line with our BMI-specific subgroup analysis we identified that extreme adherence to U-PDI was only remained significant among individuals with normal BMI. As well, after pooling the ES of studies controlling BMI as a confounder, the association between extreme adherence to U-PDI and higher odds of MetS attenuated, but remained statistically significant. It is well accepted that obesity and sedentary lifestyle and smoking are the major underlying risk factors for MetS (1, 2). These risk factors can lead to an increase in the risk of metabolic syndrome through disturbances in cardiometabolic pathways and hormone secretion, such as disturbances in the function of adipose tissue secretion (2, 46). Our included studies showed that individuals who had greater adherence to U-PDI were more likely to be smoker, had higher BMI, and a lower level of physical activity (23–25). Therefore, it seems that the observed rise in prevalence of the aforementioned risk factors among overweight and obese individuals who adhere to U-PDI may provide a rationale for the absence of a significant association between solely extreme adherence to U-PDI and odds of MetS. It is important to note that caution should be taken when interpreting this finding due to the limited number of studies considered. Additional research is required to thoroughly investigate this outcome of our study.

According to our analysis of the association between U-PDI and MetS components, extreme adherence to U-PDI was significantly associated with higher odds of MetS components, including, obesity, hypertriglyceridemia, low HDL-C, and elevated FBS. There are several different pathways by which U-PDI is associated with the increased odds of MetS components. Likewise, excessive consumption of added sugar derived from unhealthy plant foods could affect weight gain, lipid metabolism, and glycemic control (16). In addition, greater adherence to U-PDI is defined by a more intake of foods with higher glycemic index and glycemic load (refined grains, potatoes, fruit juices, sugar-sweetened beverages, and sweets and desserts) which is proposed to be associated with an increased risk of obesity, abnormal glucose homeostasis, and lipid profile (7, 17–19). Furthermore, greater adherence to U-PDI is associated with a higher intake of SFA, TFA, and lower consumption of nutrient antioxidants (vitamins C, and E, carotenoids, potassium, calcium, etc.) which may result in endothelial dysfunction, increased risk of hypertension and their consequences (24, 47–49).

### Strength and limitation

Our study has some strengths and limitations. The first strength of our study is the use of subgroup analysis to determine whether the age and gender of participants could affect the association between adherence to PDI and the risk of MetS. Secondly, this is the first systematic review and meta-analysis study investigating the association between 3 types of plant-based dietary index and the risk of MetS and its components. Additionally, the majority of included studies in this meta-analysis used the same method to assess adherence to PDIs (20), and the food frequency questionnaires used in these studies have been validated and shown to be a valuable tool for assessing habitual dietary intake. The most limitation of this study is the lack of the relevant number of studies and low sample size.

## Conclusion

Our findings revealed additional evidence regarding the favorable effects of adherence to plant-based dietary patterns on odds of MetS. Also, U-PDI rich in unhealthy carbohydrates was associated with a higher risk of MetS and its components. We can conclude that the food choices and the quality of vegetables group is as important as the quantities of vegetables in the context of a healthy dietary pattern. More studies with the higher number of participants and wider demographic diversity are needed to allow us to generalize these findings.

## Data availability statement

The data analyzed in this study is subject to the following licenses/restrictions: the data that support the findings of this study are not openly available due to reasons of sensitivity and are available from the corresponding author upon reasonable request. Requests to access these datasets should be directed to matina_844@yahoo.com.

## Author contributions

AN: Formal analysis, Methodology, Writing – original draft. EE: Data curation, Investigation, Writing – original draft. JR: Methodology, Writing – original draft. NR: Data curation, Writing – original draft. MG: Conceptualization, Supervision, Writing – review & editing.
