# Supplementary material for: The association between plant-based diet indices and metabolic syndrome: a systematic review and dose–response meta-analysis
Source: Front Nutr. 2024 Jan 8;10:1305755. doi: 10.3389/fnut.2023.1305755 (PMC10800435; doi:10.3389/fnut.2023.1305755)
Supplement: Supplementary file 1 [file Data_Sheet_1.docx]

**Supplementary file description:**

Search strategy used in this study is shown in supplementary table 1 which describes the key terms used to search in each database. Meta-regression is applied to find the potential source of heterogeneity based on the age of the participants (Supplementary Fig 1). Meta-regression is applied to find the potential source of heterogeneity based on the body mass index of the participants (Supplementary Fig 2). Funnel plot is used to assess the potential role of publication bias based on visual or analytical detection of asymmetry (Supplementary Fig 3).

**Supplementary table 1.** The structure of key terms used to search international databases (Search date: 09/25/2023)

**Supplementary Figure 1.** Meta-regression plots of the association between plant-based diet indices and metabolic syndrome based on the age of participants.

1. Overall plant-based diet index and metabolic syndrome
2. Healthy plant-based diet index and metabolic syndrome
3. Unhealthy plant-based diet index and metabolic syndrome

**Supplementary Figure 2.** Meta-regression plots of the association between plant-based diet indices and metabolic syndrome based on the BMI of participants.

1. Overall plant-based diet index and metabolic syndrome
2. Healthy plant-based diet index and metabolic syndrome
3. Unhealthy plant-based diet index and metabolic syndrome

**Supplementary Figure 3.** Funnel plot to assess publication bias

1. Overall plant-based diet index and metabolic syndrome
2. Healthy plant-based diet index and metabolic syndrome
3. Unhealthy plant-based diet index and metabolic syndrome

**Supplementary Table 1.** The structure of key terms used to search international databases (Search date: 11/30/2023)

| PubMed | Scopus | Web of Science |
| --- | --- | --- |
| (“plant based diet*”[tiab] OR “plant-based diet*”[tiab]) AND (“metabolic syndrome”[MeSH Terms] OR “metabolic syndrome”[tiab] OR “Mets”[tiab] OR “metabolic X syndrome”[tiab] OR “metabolic cardiovascular syndrome”[tiab] OR “reaven syndrome X”[tiab] OR “cardiometabolic syndrome”[tiab]) AND (2016/01/01:2023/11/30[Date - Publication]) | TITLE-ABS-KEY(“plant based diet*” OR “plant-based diet*”) AND TITLE-ABS-KEY(“metabolic syndrome” OR “Mets” OR “metabolic X syndrome” OR “metabolic cardiovascular syndrome” OR “reaven syndrome X” OR “cardiometabolic syndrome”) AND ( ( PUBYEAR > 2015 AND PUBYEAR < 2023 ) OR PUBDATETXT ( "January 2023" ) OR PUBDATETXT ( "February 2023" ) OR PUBDATETXT ( "March 2023" ) OR PUBDATETXT ( "April 2023" ) OR PUBDATETXT ( "May 2023" ) OR PUBDATETXT ( "June 2023" ) OR PUBDATETXT ( "July 2023" ) OR PUBDATETXT ( “August 2023" ) OR PUBDATETXT ( "September 2023" ) OR PUBDATETXT ( "October 2023" ) OR PUBDATETXT ( "November 2023" )) | TS=(“plant based diet*” OR “plant-based diet*”) AND TS=(“metabolic syndrome” OR “Mets” OR “metabolic X syndrome” OR “metabolic cardiovascular syndrome” OR “reaven syndrome X” OR “cardiometabolic syndrome”) AND PY=(2016-2023) |

**Supplementary Figure 1.** Meta-regression plots of the association between plant-based diet indices and metabolic syndrome based on the age of participants.

1. Overall plant-based diet index and metabolic syndrome

1. Healthy plant-based diet index and metabolic syndrome

1. Unhealthy plant-based diet index and metabolic syndrome

**Supplementary Figure 2.** Meta-regression plots of the association between plant-based diet indices and metabolic syndrome based on the BMI of participants.

1. Overall plant-based diet index and metabolic syndrome

1. Healthy plant-based diet index and metabolic syndrome

1. Unhealthy plant-based diet index and metabolic syndrome

**Supplementary Figure 3.** Funnel plot to assess publication bias

1. Overall plant-based diet index and metabolic syndrome

1. Healthy plant-based diet index and metabolic syndrome

1. Unhealthy plant-based diet index and metabolic syndrome
